# A micro-beamstop with transmission detection by fluorescence for scanning-beam synchrotron scattering beamlines

**DOI:** 10.1107/S1600576724009129

**Published:** 2024-10-29

**Authors:** Henrik Birkedal, Michael Sztucki, Moritz Stammer, Anastasiia Sadetskaia, Manfred C. Burghammer, Tilman A. Grünewald

**Affiliations:** a Department of Chemistry and iNANO Aarhus University 14 Gustav Wieds Vej 8000Aarhus Denmark; b European Synchrotron Radiation Facility (ESRF) Avenue des Martyrs 71 Grenoble38000 France; c Aix-Marseille Univ., CNRS, Centrale Med, Institut Fresnel Marseille France; Montanuniversität Leoben, Austria

**Keywords:** X-ray absorption, scanning SAXS/WAXS, nanoprobes, data correction, avalanche photodiodes

## Abstract

Quantitative X-ray diffraction approaches require careful correction for sample transmission. Though this is a routine task at state-of-the-art small-angle X-ray scattering (SAXS), wide-angle X-ray scattering (WAXS) or diffraction beamlines at synchrotron facilities, the transmission signal cannot be recorded concurrently with SAXS/WAXS when using the small, sub-millimetre beamstops at many X-ray nanoprobes during SAXS/WAXS experiments due to the divergence-limited size of the beamstop and the generally tight geometry. This is detrimental to the data quality and often the only solution is to re-scan the sample with a PIN photodiode as a detector to obtain transmission values. In this manuscript, we present a simple yet effective solution to this problem in the form of a small beamstop with an inlaid metal target for optimal fluorescence yield. This fluorescence can be detected with a high-sensitivity avalanche photodiode and provides a linear counter to determine the sample transmission.

## Abstract 2

The correct determination of X-ray transmission at X-ray nanoprobes equipped with small beamstops for small- and wide-angle X-ray scattering collection is an unsolved problem with huge implications for data correction pipelines. We present a cost-effective solution to detect the transmission via the X-ray fluorescence of the beamstop with an avalanche photodiode.

## 1. Introduction

For most quantitative data analysis approaches of X-ray scattering patterns, normalizing the intensity is a key element of the data analysis pipeline. Although establishing a ‘correct’ data correction pipeline is a non-trivial task in itself (Pauw, 2013 ▸), it is clear that at least two fundamental parameters need to be known, the incoming beam flux and the X-ray transmission of the sample. Note that for heterogeneous samples sampled in scanning experiments (Grünewald *et al.*, 2016*a* ▸; Grünewald *et al.*, 2016*b* ▸; Hauge Bünger *et al.*, 2006 ▸; Wittig & Birkedal, 2022 ▸; Gourrier *et al.*, 2010 ▸; Pabisch *et al.*, 2013 ▸; Flatscher *et al.*, 2024 ▸; Karner *et al.*, 2022 ▸) the transmission varies spatially and in itself provides important information on the sample. At synchrotron sources, a certain fluctuation of incident intensity is inevitable, making online monitoring of the incident beam intensity essential; it is routinely carried out with ionization chambers or transmission photodiodes (for higher energies). Their use is well established for state-of-the-art small-angle X-ray scattering (SAXS) or diffraction beamlines (Narayanan *et al.*, 2022 ▸; Smith *et al.*, 2021 ▸). However, for spatially resolved studies, nanofocusing makes it challenging to place ionization chambers between the focusing optics and the sample, although compact solutions have been adapted (Kocsis & Somogyi, 2003 ▸) and comparatively long working distance optics like KB mirrors or multi-layer Laue lenses offer sufficient focal length to accommodate them.

The most pressing issue is the characterization of the sample absorption properties to enable appropriate corrections. These corrections range from simple background subtraction for 2D scanning experiments to more exigent corrections for 3D tomography techniques. For example, proper self-absorption correction in XRF tomography requires knowledge of the sample composition and absorption behaviour (Wittig *et al.*, 2019 ▸; Palle *et al.*, 2020 ▸). Small-angle scattering tensor tomography needs a good transmission signal for the correct scaling of backprojected intensities (Liebi *et al.*, 2015 ▸; Nielsen *et al.*, 2023 ▸). This is even more crucial for small- and wide-angle X-ray scattering (SAXS/WAXS) tensor tomography (Grünewald *et al.*, 2020 ▸, 2023 ▸). Texture tomography (Frewein *et al.*, 2024 ▸) requires correction not only for the absorption of the direct transmitted beam but also for the absorption of the diffracted radiation through the sample. A correction scheme has been implemented for this by Grünewald *et al.* (2023 ▸), but it obviously requires knowledge of the 2D absorption properties of the sample and the reconstructed 3D absorption volume of the sample.

Frequently, a transmission measurement is carried out by direct detection of the X-rays with photodiodes embedded into the beamstop (BS) (Smith *et al.*, 2021 ▸; Narayanan *et al.*, 2022 ▸). The BS is in this approach typically placed just before the detector. The X-ray beam is of low divergence and focused onto the detector/BS. These BSs can be few millimetres large to accommodate a photodiode and shield to avoid X-ray leakage onto the detector. As the photodiode is usually hit by the direct beam, this leads to lifetime issues of the photodiode. An interesting approach is to use the fluorescence of a BS for the detection to reduce the dose. A successful implementation of this strategy at the P12 beamline at PETRA III (DESY) was reported by Blanchet *et al.* (2015 ▸). For X-ray micro- and nanoprobes that carry out combined SAXS and WAXS experiments (SAXS/WAXS) such as ID13 at the European Synchrotron Radiation Facility (ESRF), µSpot at Bessy II, NanoMAX and ForMAX at MAX-IV, or P06 at Petra III to name just a few, direct detection is more problematic: the X-ray beams have higher divergence and are obviously focused on the sample, not the detector, position. The need for an optimized background to deal with thin samples, possibly of weakly scattering biological origin, calls for a BS close to the sample in conjunction with a flight tube to eliminate air scattering. Concurrently, the need for a small *q*_min_ to access SAXS information dictates that the BS be very small, optimally just matching the beam divergence at the BS position. In practice, such BSs are 200–500 µm in diameter and are made from lead, gold or other highly absorbing materials to avoid parasitic BS diffraction. Generally, space is at a premium in these kinds of setups and adding an extra element to detect the transmission is generally not possible.

The small BS size prohibits integration of photodiodes and the inherent fragility and damage-prone location of a BS requires a solution that is easy and cheap to replace. One approach is to use a semi-transparent BS where the BS stops most of the incident X-rays, letting only an attenuated fraction pass that is within the linear counting rate of the detector. However, such a BS needs to be carefully tailored to attain the required ∼10^−6^ transmission and inherently suffers from higher-harmonic high-energy pollution of the X-ray beam. Thus, semi-transparent BSs are mostly attractive solutions for high-energy beamlines where higher harmonics have significantly higher energy than the main beam and are practically not absorbed at all by the detector material.

**Figure 1:**
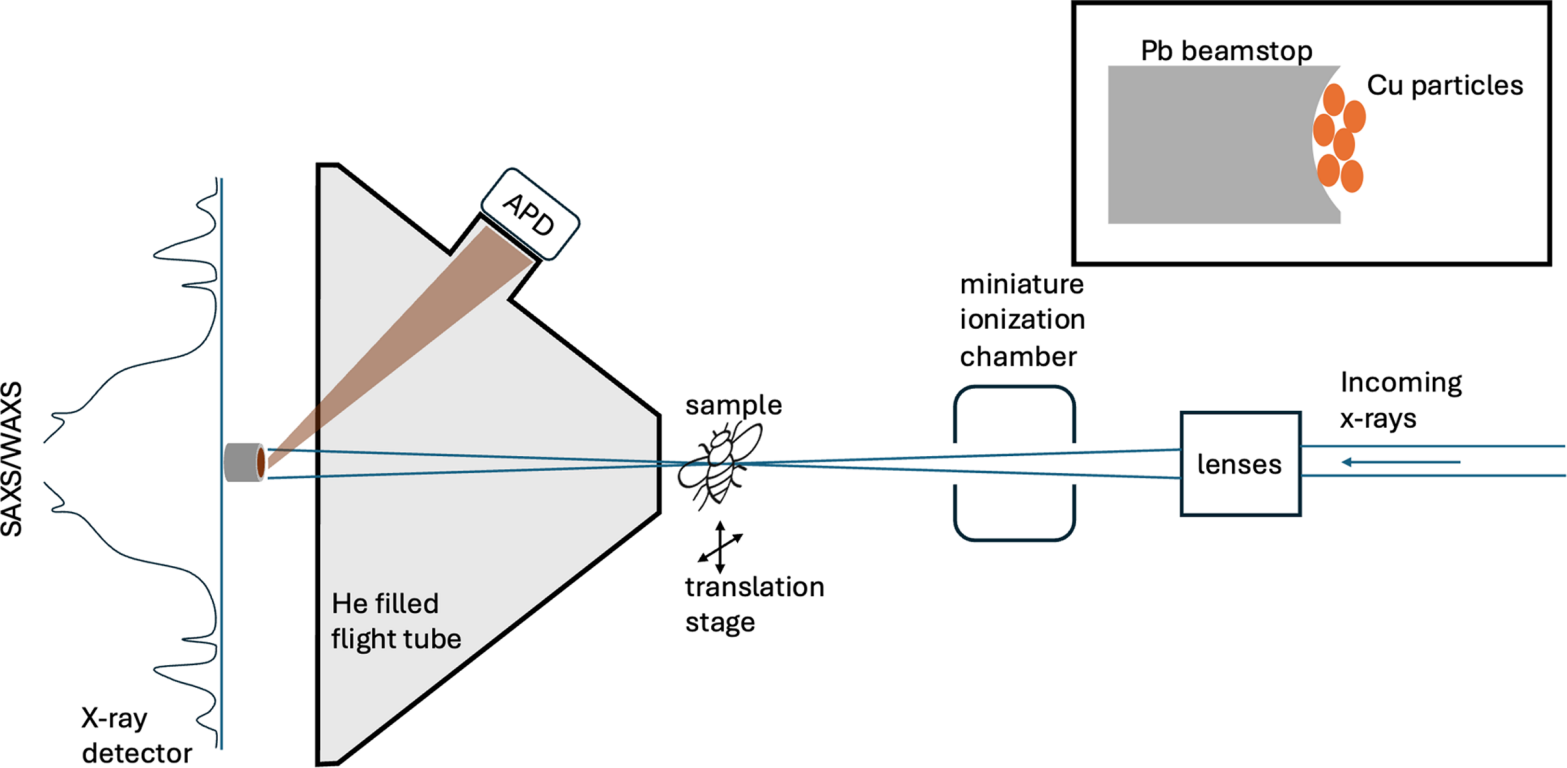
Schematic setup of an X-ray nanoprobe for SAXS/WAXS experiments. The incoming beam is focused by X-ray lenses onto the sample. A miniature ionization chamber detects the incoming beam flux. The diverging, transmitted X-ray beam is blocked by a small (∼250 µm) BS and only the sample SAXS and WAXS signal is recorded by the detector. The BS is filled with copper powder and the emitted Cu *K* fluorescence is detected by an APD. A sketch of the BS is shown in the inset.

Many nanoprobes, however, operate at intermediate X-ray energies (10–30 keV). Therefore, we propose a simple scheme for *in situ* transmission measurement (Fig. 1 ▸) that relies on the detection of fluorescence emitted from the BS by an avalanche photodiode (APD). The APD has a comparatively small form factor, while providing excellent counting capabilities due to the strong amplification of even single-photon events (Baron *et al.*, 2006 ▸) and can be integrated into an already existing flight tube and the limited space available for SAXS/WAXS nanoprobe experiments (see Fig. S1 of the supporting information). We present the design, implementation and performance characteristics of a low-cost, efficient, fluorescence based micro-BS transmission detection scheme. We also discuss avenues for further improvement of performance.

## 2. Materials and methods

The BS was fabricated from 250 µm lead wire (99.5% purity, GoodFellow). A 2 mm-long piece was straightened out and rolled to create a slight burr on the edge, effectively forming a pit. This pit in the front of the BS was enlarged using a small tungsten needle to about 100 µm diameter and filled with copper powder (98%, 10–25 µm particle size, Sigma–Aldrich) that was fixed in place with a microdrop of superglue, applied with an eyelash. The size of the pit was chosen to match the beam size while still providing enough material around the fluorescent target to stop any leakage of fluorescence and scattering/diffraction onto the diffraction detector. The BS was mounted on a glass capillary, positioned 60 mm downstream of the sample, just behind a flight tube described in more detail below, and aligned in the X-ray beam of ID13, ESRF. Note that it is important to embed the fluorescence target well within the pit of the BS to avoid parasitic scattering or fluorescence leaking in the X-ray diffraction detector.

X-ray SAXS/WAXS experiments were carried out at the EH3 nanobranch of ID13. An energy of 9.808 keV was selected with a channel-cut Si(111) monochromator from a U35 undulator and focused on the sample position with a set of crossed multilayer Laue lenses (MLLs) (Niese *et al.*, 2014 ▸). With a pre-focusing scheme, this yielded a flux of ∼2 × 10^9^ photons s^−1^ in a ∼300 × 300 nm-sized beam. This particular set of lenses produced a beam with 36 mm focal length and a divergence of 2 mrad. A square 40 µm order selection aperture was employed to clean the beam. This aperture was positioned 1.5 mm upstream of the sample to minimize the air background. In principle, a working distance as large as ∼5 mm can be realized at this energy and even more at higher energies. The diffracted X-rays were collected using an Eiger 4M detector positioned 80 mm downstream of the sample. The incoming beam flux was registered using a mini-ionization chamber (Kocsis & Somogyi, 2003 ▸), placed between the MLL optics and an order selection aperture. The induced current was read out using an ESRF-built electrometer (MoCo box; https://www.esrf.fr/Instrumentation/DetectorsAndElectronics/moco).

A 3D-printed helium-filled flight tube (60 mm length, 120 × 120 mm exit window size) with a 10 × 10 mm, 1 µm-thick Si_3_N_4_ entry window (Norcada) and a 4 µm polypropyl­ene (PP) foil (Sigma Aldrich) exit window was used. The flight tube had a port to mount an APD and a similar PP window was used to seal this port. This geometry allowed us to observe the BS at an angle of 70° and a distance of 60 mm. The APD (Perkin-Elmer C30703, 10 × 10 mm active surface, 110 µm thickness) was biased at 325 V and read out using the ESRF-built APD controller electronic (‘ACE’) readout module (Baron *et al.*, 2006 ▸) operated in integrating mode. The advantage of the APD employed is the very high dynamic range, enabling us to count from a single-photon regime up to the full direct incident beam in the 1 × 10^12^ photons s^−1^ flux range at 15 keV.

An *ex situ* transmission measurement was carried out with a conventional silicon PIN photodiode (19 mm diameter active area, 500 µm thickness, Canberra 300–500CB), detecting the direct X-ray beam. This diode was placed 800 mm downstream of the samples and the induced current was read using an ESRF-built current amplifier in the monochromator control box (MoCo).

**Figure 2:**
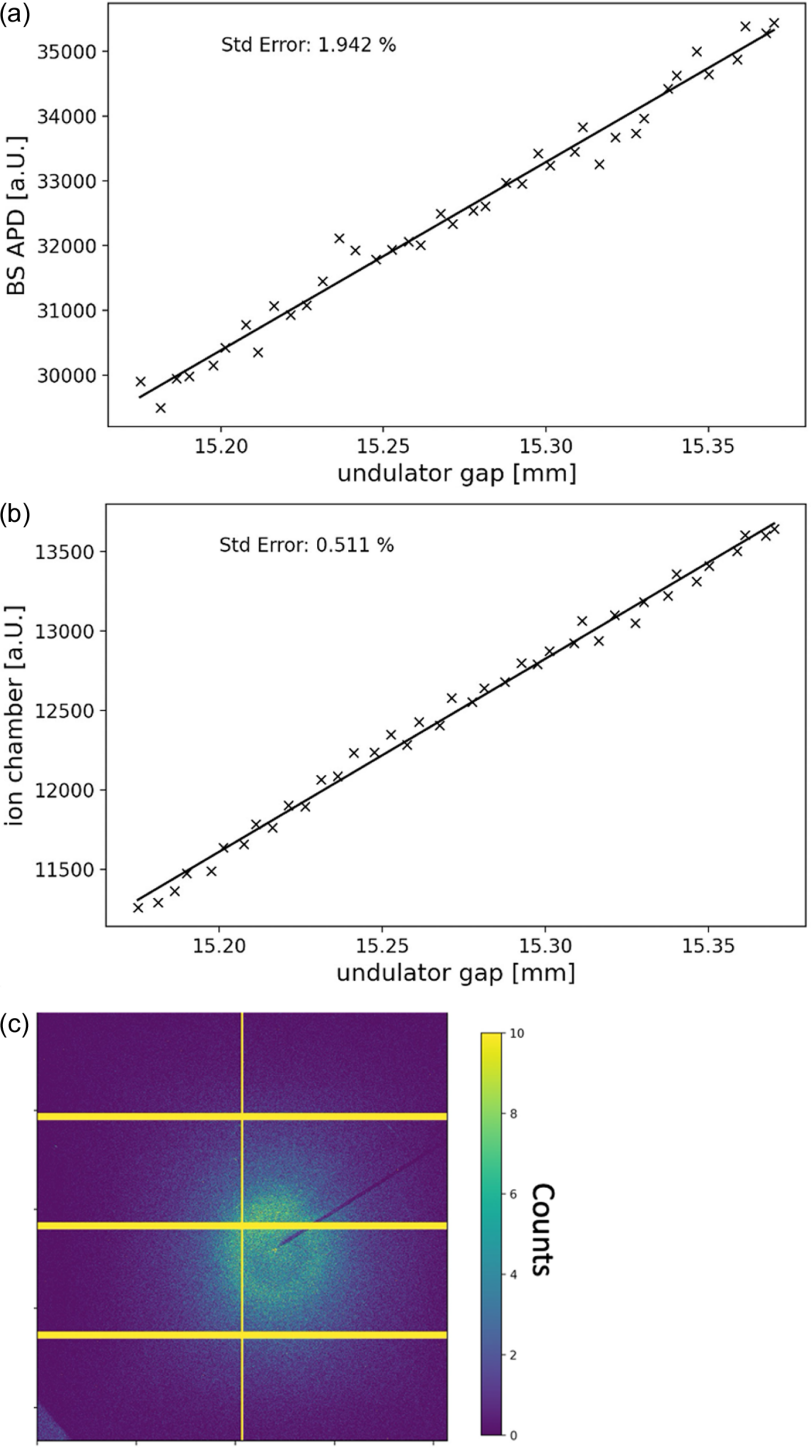
Linearity scan comparing (*a*) the BS APD and (*b*) the ion chamber. The normalized standard error of the BS APD was determined to be 1.942% in contrast to 0.511% for the ion chamber. (*c*) Background image with 1 s exposure time.

For a linearity check, the undulator was scanned over a range of 0.2 mm with an exposure time of 0.1 s and the resulting BS fluorescence signal was compared with that from the PIN diode. A first-order polynomial was fitted to the data and the normalized standard error calculated. The results are presented in Fig. 2 ▸. The range of the linearity check was chosen to mimic the expected range of absorption within a heterogenous sample.

X-ray absorption scans of a thin slice (20 µm) of osteonal cow bone from a butcher were carried out with a step size of 1 µm and an exposure time of 1 s for both the BS and the photodiode transmission approach. With the BS, X-ray diffraction patterns were also collected. The accessible *q*-range was 0.15–35 nm^−1^, giving access to collagen diffraction as well as mineral particle SAXS and diffraction signal. Dark currents of 118.3 counts/0.1 s for the BS and 3.3 counts/0.1 s for the photodiode were used. The ion chamber presented 0 counts dark current, which was subtracted for each counter, and the ionization chamber was used to normalize the incoming beam intensity. We underline that the counts here are not physical single-photon counts but rather the digitized counts from the current detection of the counting card. Transmission was calculated with reference to empty air measurements.

## 3. Results and discussion

We tested the BS APD setup (Fig. 1 ▸) under low-flux conditions (∼2 × 10^9^ photons s^−1^ in ∼300 × 300 nm at 9.808 keV) to benchmark the performance under challenging conditions. Fig. 2 ▸ shows a comparison of the linearity of the BS APD and the ion chamber setups obtained by the undulator scan. A normalized standard error of 1.942% was observed for the BS, in contrast to 0.511% for the ion chamber. Fig. 2 ▸(*c*) shows the background diffraction pattern for a 1 s exposure time. No pronounced diffraction or fluorescence signal from the BS can be detected. This is a good starting point for further optimization and can be expected to improve significantly at the higher photon fluxes most often used, *e.g.* at ID13 where the U18 undulator provides ∼10^12^ photons s^−1^ in the 200 × 200 nm beam at 15 keV.

**Figure 3:**
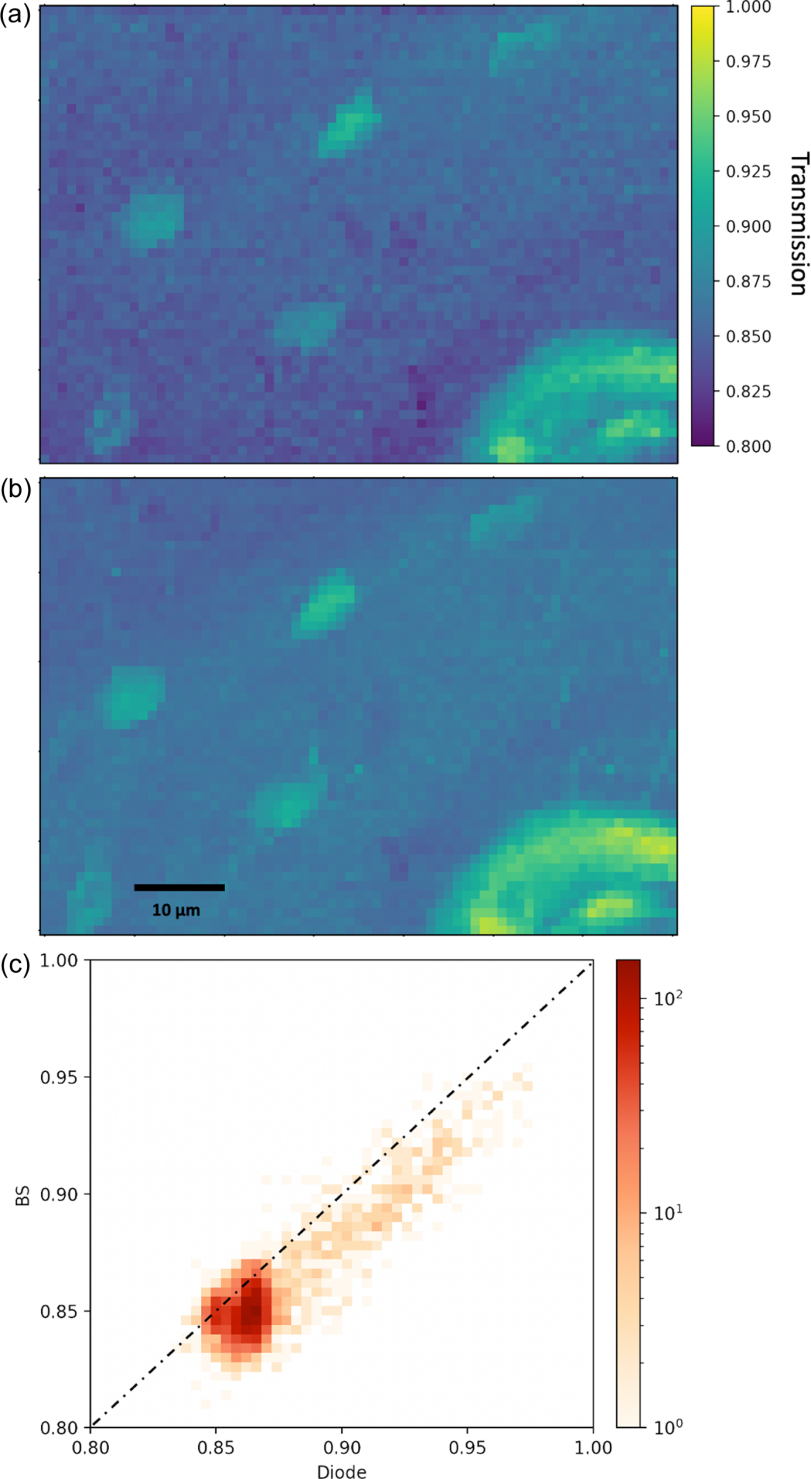
Transmission maps obtained by (*a*) the BS APD and (*b*) the PIN photodiode, showing very similar transmission values and a slightly higher noise level for the BS APD. (*c*) Transmission of the BS APD and photodiode in a bivariate histogram; the comparison of the BS APD and diode unveils a slight offset of the diode to higher transmission values. This stems from the additional SAXS signal detected by the diode.

To characterize the transmission performance, a slice of cow bone was scanned both with the BS APD [Fig. 3 ▸(*a*)] and, after removal of the BS, with a photodiode [Fig. 3 ▸(*b*)]. Osteocyte lacunae (Rodriguez-Palomo *et al.*, 2023 ▸; Wittig & Birkedal, 2022 ▸) and a large blood vessel (lower right) can be seen in regions with transmission reaching up to 0.9 whereas the bulk of the bone is around 0.8. The maps show similar features with the BS APD measurement appearing slightly noisier.

Fig. 3 ▸(*c*) compares the BS APD and photodiode in a bivariate histogram, demonstrating a clear linear correlation. The photodiode values are, however, offset towards larger transmission readings relative to the BS APD. We interpret this as resulting from the fact that the two counters record different signals. The BS APD records mostly the transmitted direct beam through its interaction with the BS in the form of fluorescence. In contrast, the photodiode with its larger area additionally detects the SAXS signal from the sample, thereby systematically underestimating the transmission for strongly scattering samples such as bone. This highlights that careful analysis of the recorded signal is necessary.

## 4. Conclusions

We presented an easy and straightforward way to measure the transmission signal via the fluorescence emitted from a small BS and collected via an APD. This requires only the implementation of an additional APD. For the current case, we chose copper powder with an excitation energy of 8.98 keV to create fluorescence in a 250 µm-diameter lead BS as the excitation energy of 13.04 keV for lead *L*-edge emission is too low. We have successfully tested BSs based solely on lead fluorescence at 15 keV. Carefully tailoring the BS material (like Fe, Au, Pb) to the target energy is a promising strategy as each of these materials can be obtained as high-purity wires. This setup enabled a detection of the large *q*-range 0.15–35 nm^−1^ on the Eiger 4M detector. An additional advantage of the BS APD setup is that the APD settings can be used even when changing the incident wavelength since the measured fluorescence energy and the response of the APD are constant.

Further development should be directed at increasing the detection efficiency of the emitted fluorescence, either by increasing the solid angle covered by the APD or by using multiple APDs. The current design with a high take-off angle aims at reducing the scattering signal from the BS and favours the fluorescence emission, but one could equally optimize for the former. Here, considerations on the employed grain size of the fluorescing material also come into play. Another question is whether an APD is required for efficient detection or if a conventional PIN diode with a high-gain amplifier can deliver a similar performance with a further reduced form factor and not requiring the high-voltage biasing of the diode, which can be a disadvantage for electromagnetically sensitive environments. Furthermore, noise reduction along the detection chain (mostly the APD readout) will likely reduce the noise floor significantly.

Accurate scanning-based SAXS/WAXS nanoprobes hinge on the accurate detection of the transmission and the associated corrections. This development presents a solution to this pressing problem and will enable significant advancement for the data treatment of scanning diffraction or tomography data obtained at nanoprobes.

## Supplementary Material

Supporting figure. DOI: 10.1107/S1600576724009129/xx5056sup1.pdf
