# Supplementary material for: A micro-beamstop with transmission detection by fluorescence for scanning-beam synchrotron scattering beamlines
Source: J Appl Crystallogr. 2024 Oct 29;57(Pt 6):2043–7. doi: 10.1107/S1600576724009129 (PMC11611290; doi:10.1107/S1600576724009129)
Supplement: Supplementary file 1 [file j-57-02043-sup1.pdf]

JOURNAL OF APPLIED CRYSTALLOGRAPHY

Volume 57 (2024)

Supporting information for article:

A micro-beamstop with transmission detection by fluorescence for scanning-beam synchrotron scattering beamlines

Henrik Birkedal, Michael Sztucki, Moritz Stammer, Anastasiia Sadetskaia, Manfred C. Burghammer and Tilman A. Grünewald

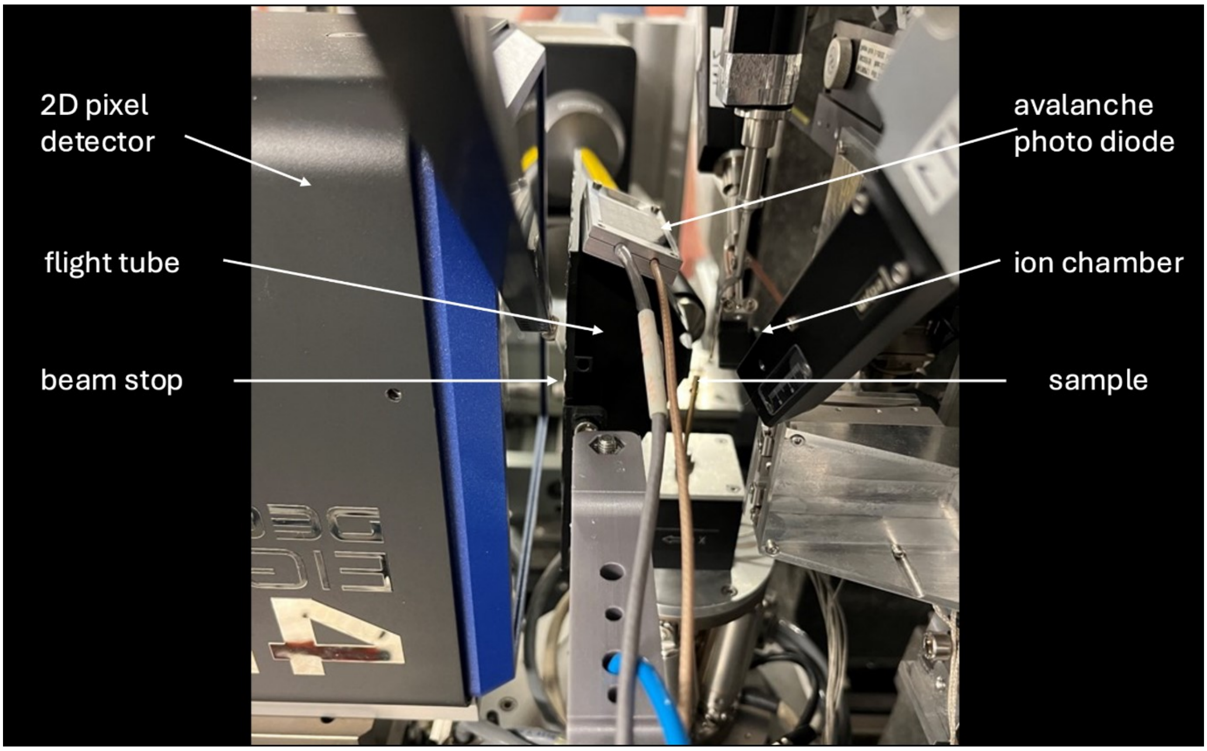

Figure S1 SAXS/WAXS setup at ID13 with implemented BS APD detection. The photograph has been digitally mirrored to have the beam enter from the right. Text on the photograph therefore appears mirrored.
